# Structural and kinetic studies on RosA, the enzyme catalysing the methylation of 8‐demethyl‐8‐amino‐d‐riboflavin to the antibiotic roseoflavin

**DOI:** 10.1111/febs.13690

**Published:** 2016-03-17

**Authors:** Chanakan Tongsook, Michael K. Uhl, Frank Jankowitsch, Matthias Mack, Karl Gruber, Peter Macheroux

**Affiliations:** ^1^ Institute of BiochemistryGraz University of TechnologyAustria; ^2^ Institute of Molecular BiosciencesUniversity of GrazAustria; ^3^ Department of BiotechnologyInstitute for Technical MicrobiologyMannheim University of Applied SciencesGermany

**Keywords:** antibiotic, crystallography, enzyme kinetics, *S*‐adenosylmethionine, transferase

## Abstract

*N*,*N*‐8‐demethyl‐8‐amino‐d‐riboflavin dimethyltransferase (RosA) catalyses the final dimethylation of 8‐demethyl‐8‐amino‐d‐riboflavin (AF) to the antibiotic roseoflavin (RoF) in *Streptomyces davawensis*. In the present study, we solved the X‐ray structure of RosA, and determined the binding properties of substrates and products. Moreover, we used steady‐state and rapid reaction kinetic studies to obtain detailed information on the reaction mechanism. The structure of RosA was found to be similar to that of previously described *S*‐adenosylmethionine (SAM)‐dependent methyltransferases, featuring two domains: a mainly α‐helical ‘orthogonal bundle’ and a Rossmann‐like domain (α/β twisted open sheet). Bioinformatics studies and molecular modelling enabled us to predict the potential SAM and AF binding sites in RosA, suggesting that both substrates, AF and SAM, bind independently to their respective binding pocket. This finding was confirmed by kinetic experiments that demonstrated a random‐order ‘bi‐bi’ reaction mechanism. Furthermore, we determined the dissociation constants for substrates and products by either isothermal titration calorimetry or UV/Vis absorption spectroscopy, revealing that both products, RoF and *S*‐adenosylhomocysteine (SAH), bind more tightly to RosA compared with the substrates, AF and SAM. This suggests that RosA may contribute to roseoflavin resistance in *S. davawensis*. The tighter binding of products is also reflected by the results of inhibition experiments, in which RoF and SAH behave as competitive inhibitors for AF and SAM, respectively. We also showed that formation of a ternary complex of RosA, RoF and SAH (or SAM) leads to drastic spectral changes that are indicative of a hydrophobic environment.

**Database:**

Structural data are available in the Protein Data Bank under accession number 4D7K.

**Abbreviations**
AF: 8‐demethyl‐8‐amino‐d‐riboflavin
ITC: isothermal titration calorimetry
MAD: multiple‐wavelength anomalous dispersion
MAF: 8‐demethyl‐*N*‐8‐methylamino‐d‐riboflavin
RoF: roseoflavin
RosA: 8‐demethyl‐*N*,*N*‐8‐amino‐d‐riboflavin dimethyltransferase
SAH: *S*‐adenosylhomocysteine
SAM: *S*‐adenosylmethionine

## Introduction

**Scheme 1:**
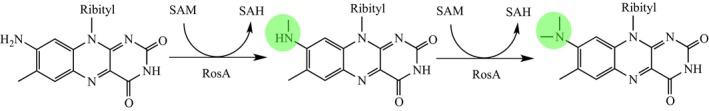
The catalytic reaction of RosA.

The Gram‐positive soil bacteria *Streptomyces davawensis* and *Streptomyces cinnabarinus* produce roseoflavin (RoF), the only known natural riboflavin (vitamin B_2_) analogue with antibiotic activity. In contrast to most other antibiotics, RoF has multiple cellular targets and negatively affects flavoproteins as well as FMN riboswitches 1, 2, 3, 4, 5, 6, 7, 8. RoF may be considered a natural anti‐metabolite, and was postulated to be biosynthesized from riboflavin through 8‐demethyl‐8‐amino‐d‐riboflavin (AF) and 8‐demethyl‐8‐methylamino‐riboflavin (MAF) 9, 10. The occurrence of the intermediates AF and MAF was confirmed by the discovery of the first enzyme of RoF biosynthesis, the *S*‐adenosylmethionine (SAM)‐dependent dimethyltransferase *N*,*N*‐8‐demethyl‐8‐amino‐d‐riboflavin dimethyltransferase (RosA) 11. This enzyme synthesizes RoF from AF (via MAF) in two consecutive methylation reactions, as shown in Scheme 1. The corresponding gene (*rosA*) is present in a cluster comprising ten genes. The remaining genes of this cluster were found not to be involved in RoF synthesis, and it is presently unclear which genes/enzymes are responsible for synthesis of the key intermediate AF 12.

The important role of RosA for completion of RoF biosynthesis prompted us to determine the three‐dimensional structure of this enzyme and to investigate the mechanism of the consecutive methylation reactions in more detail. We show that RosA has a topology similar to other SAM‐dependent methyltransferases, containing a mainly α‐helical ‘orthogonal bundle domain’ and a Rossmann‐like fold. Determination of the three‐dimensional structure also enabled us to locate the binding sites for the substrates AF and SAM. In addition, we performed steady‐state and rapid reaction studies, and determined the dissociation constants for the substrates of RosA as well as for the products. It was found that AF and SAM bind independently to RosA according to a random‐order mechanism. The products of the RosA reaction, RoF and SAH, exhibit higher affinity for the protein than the substrates AF and SAM do, leading to competitive product inhibition. RosA is a comparably slow enzyme, with a turnover rate (*k*_cat_) of 0.06 min^−1^ for the overall dimethylation reaction. As RoF is a more potent antibiotic than AF and MAF, the slow synthesis of RoF and the tight binding of products may contribute to protection of the strains *S. davawensis* and *S*. *cinnabarinus* from the antibiotic that they produce.

## Results

### Crystal structure of RosA

Initial attempts to solve the structure of RosA by molecular replacement were unsuccessful despite a thorough search for appropriate template structures using the online tools phyre2 13, fugue 14 and caspr 15. Therefore, RosA labelled with selenomethionine (SeMet) (seven methionine residues in 353 amino acids) was produced, and the structure was solved by multiple‐wavelength anomalous dispersion (MAD) using a single tetragonal crystal (Table 1). The structure (one protomer in the asymmetric unit) was partially refined to a resolution of 3.5 Å, and this model was then used to solve the structure of a triclinic crystal form (space group *P*1) by molecular replacement. This crystal contained six molecules per asymmetric unit, and the structure was refined at a resolution of 2.2 Å, yielding final values of *R* = 20% and *R*_free_ = 25% (Table 2).

**Table 1:** Statistics for the SeMet MAD datasets. Values in parentheses are for the highest‐resolution shell

	Peak	Inflection	Remote
X‐ray source	SLS X06DA‐PXIII		
Wavelength (Å)	0.9791	0.9796	0.9714
Temperature (K)	100	100	100
Space group	*I*4_1_22	*I*4_1_22	*I*4_1_22
Cell dimensions			
*a* = *b*,* c* (Å)	112.54, 132.27	112.46, 132.48	112.60, 132.27
Resolution (Å)	47.04–3.35 (3.53–3.35)	47.02–3.54 (3.60–3.54)	47.06–3.42 (3.73–3.42)
Total number of reflections	166 043 (22 355)	141 532 (20 491)	156 742 (21 751)
Unique number of reflections	6400 (905)	5454 (771)	6031 (848)
Multiplicity	25.9 (27.7)	26.0 (26.6)	26.0 (25.6)
Anomalous multiplicity	14.2 (13.1)	14.2 (14.1)	14.2 (13.7)
Completeness (%)	99.9 (99.7)	100.0 (100.0)	99.9 (99.6)
Anomalous completeness (%)	99.9 (99.4)	99.9 (99.4)	99.9 (99.3)
*R*_p.i.m._ (%)	1.3 (5.9)	1.0 (5.7)	1.1 (6.3)
*R*_meas_ (%)	6.4 (29.5)	5.2 (29.8)	5.6 (32.2)
<I/αI)>	48.3 (12.8)	51.1 (14.8)	50.0 (12.2)
CC_1/2_	1.000 (0.989)	1.000 (0.987)	1.000 (0.989)
CC*	1.000 (0.995)	1.000 (0.995)	1.000 (0.997)

**Table 2:** Data collection and refinement statistics. Values in parentheses are for the highest‐resolution shell

	RosA (native)
Data collection	
X‐ray source	ESRF ID23‐1
Wavelength (Å)	1.000
Temperature (K)	100 K
Space group	*P*1
Cell dimensions	
*a*,* b*,* c* (Å)	82.72, 82.76, 96.45
α, β, γ (°)	95.1, 98.7, 114.5
Resolution (Å)	34.77–2.22 (2.26–2.22)
Total number of reflections	168 833 (3484)
Unique number of reflections	86 780 (1838)
Multiplicity	1.9 (1.9)
Completeness (%)	77.4 (33.0)
*R*_p.i.m._ (%)	3.1 (39.8)
*R*_meas_ (%)	4.3 (56.2)
<I/σ(I)>	12.7 (1.70)
CC_1/2_	0.999 (0.756)
CC*	1.000 (0.928)
Refinement	
*R*_work_	0.1949
*R*_free_	0.2485
Number of atoms	15 787
Protein	15 518
Water	269
B‐factors (Å^2^)	
Protein	66.1
Water	48.9
All atoms	65.8
RMSDs	
Bond lengths (Å)	0.003
Bond angles (°)	0.620
Ramachandran outliers (%)	0.15
PDB ID	4D7K

**Figure 1:**
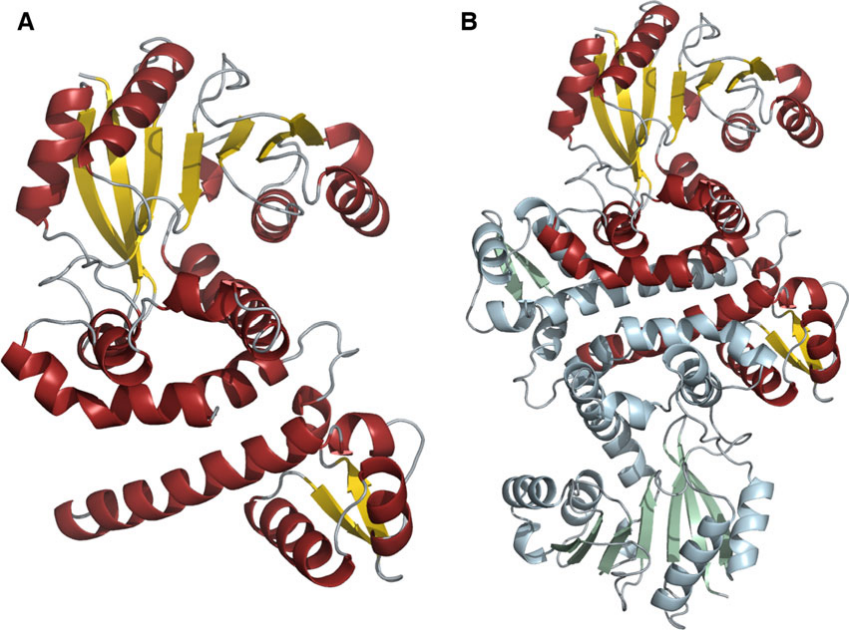
Schematic representation of the structure of RosA. (A) Cartoon representation of the RosA protomer, showing α‐helices in red, β‐sheets in yellow, and loops in grey. (B) Cartoon representation of the RosA dimer with one protomer shown in red, yellow and grey, and the other in pale blue, green and grey.

The RosA protomer comprises two domains: a mainly α‐helical ‘orthogonal bundle domain’ and a Rossmann‐like domain (α/β twisted open sheet). The orthogonal bundle (five α‐helices and one antiparallel β‐sheet) consists of residues 1–98. Residues 99–178 form an inter‐domain region (five helices) that connects the Rossmann motif to the orthogonal bundle. The Rossmann motif (residues 179–353) comprises a seven‐stranded β‐sheet core consisting of five parallel and two antiparallel β‐strands, which are connected by two pairs of α‐helices (Fig. 1A). To classify the domains, we performed a cath database (http://www.cathdb.info/) search using the CATHEDRAL algorithm 16. The orthogonal bundle belongs to the CATH superfamily ‘winged helix repressor DNA binding domain’ (CATH classification: 1.10.10.10; Sequential Structure Alignment Program (SSAP) score = 87.6; RMSD = 2.7 Å) and the functional family ‘caffeic acid 3‐*O*‐methyltransferase 1’. The Rossmann‐like domain was found to be a member of the CATH superfamily ‘Vaccinia Virus protein VP39’ (CATH classification: 3.40.50.150; SSAP score = 85.4, RMSD = 4.1 Å). The algorithm treated the inter‐domain residues as part of the Rossmann motif.

**Figure 2:**
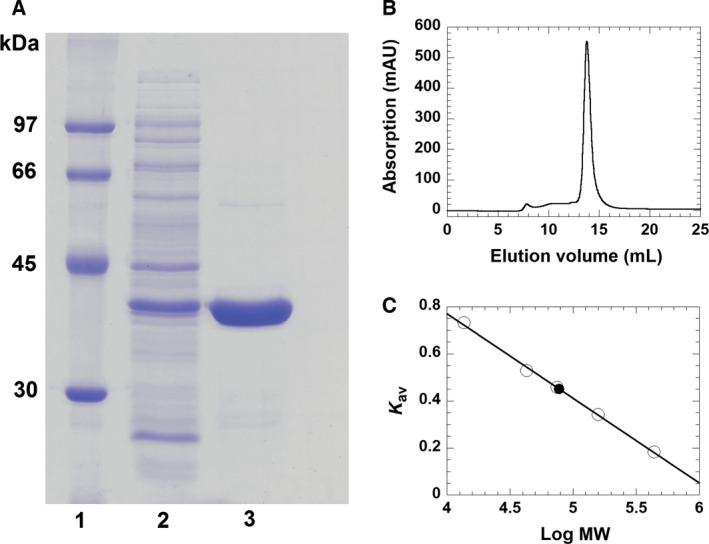
Determination of the purity and native molecular mass of RosA using SDS/PAGE and FPLC. (A) Determination of the subunit molecular mass of RosA after purification by Ni‐Sepharose FPLC was performed by SDS/PAGE (12.5%). Lane 1, low‐molecular‐mass protein marker; lane 2, crude extract; lane 3, protein fraction after purification by Ni‐Sepharose FPLC. The subunit molecular mass of RosA was estimated to be 38 kDa. (B) Determination of the native molecular mass of RosA using Superdex 200 10/300 GL chromatography. (C) Plot of the partition coefficient (*K*_av_) against the logarithm of molecular mass of standard proteins (ferritin, 440 kDa; aldolase, 158 kDa; conalbumin, 75 kDa; ovalbumin, 43 kDa; ribonuclease A, 13.7 kDa). The native molecular mass of RosA (filled circle) was estimated to be 77 kDa.

A PISA analysis 17 predicted that a dimer is the most probable oligomer of RosA in solution. This prediction was confirmed by size exclusion chromatography (Fig. 2). Dimerization occurs in a head‐to‐head arrangement (Fig. 1B), and is mediated by interactions of adjacent α‐helices of the bundle motif (residues 1–36 and residues 60–73), the inter‐domain α‐helices comprising residues 101–109 and residues 111–126, and one helix from the Rossmann motif (residues 293–305).

The RosA protomers in the asymmetric unit exhibit very similar structures. Pairwise superpositions of the Rossmann domains in the six crystallographically independent protomers yielded RMSD values in the range from 0.2 to 0.9 Å (for approximately 160 aligned Cα atoms). The same analysis yielded mean RMSD values of 0.3 and 0.6 Å for the N‐terminal and intermediate regions. The values are slightly higher for superpositions of the entire chains (1.2 Å) and the three dimers (1.6 Å).

To identify protein structures resembling RosA in terms of its tertiary structure, we performed a Dali server analysis 18. The structures with the highest *Z* scores were mitomycin 7‐*O*‐methyltransferase from *Streptomyces lavendulae* (PDB ID 3GWZ), a probable phenazine‐specific methyltransferase from *Pseudomonas aeruginosa* (PDB ID 2IP2), the CALO1 methyltransferase from *Micromonospora echinospora* (PDB ID 3LST), and a caffeic acid *O*‐methyltransferase from *Sorghum bicolor* (PDB ID 4PGH), with *Z* scores of 33, 32, 30 and 30, respectively. These proteins share 21–35% sequence identity with RosA. Superposition with the most similar structure (3GWZ, 35% sequence identity, 76% query coverage) resulted in an RMSD of 3.5 Å for the isolated subunit (317 of 324 aligned Cα atoms) and 6.6 Å for the dimers (618 of 650 aligned Cα atoms). Superposition of the Rossmann domains of RosA (residues 179–345) and 3GWZ (residues 179–349) resulted in an RMSD of 0.9 Å (118 of 160 aligned Cα atoms). This indicates that, despite an overall low sequence identity, the structure of RosA, especially the Rossmann domain, closely resembles that of other methyltransferases.

### Prediction of the SAM/SAH binding site

**Figure 3:**
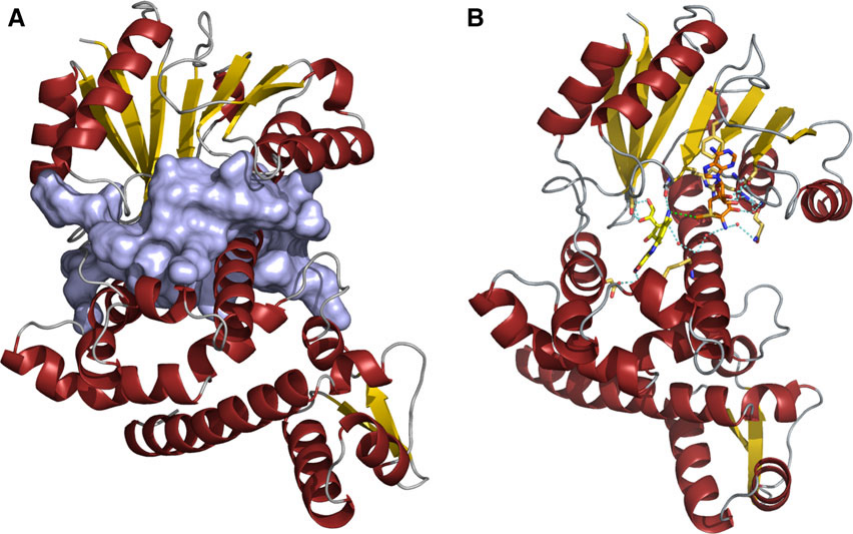
Schematic representation of the predicted binding pocket of RosA. (A) Surface and cartoon representation of the binding pocket of RosA predicted using casox 19. (B) Cartoon representation of RosA with predicted binding of AF (shown as yellow sticks) and SAM (shown as orange sticks). Potential hydrogen bonding interactions are indicated by blue dashed lines. Water molecules are represented as red spheres. The distance between the reactive methyl group of SAM and the amino group of AF is indicated by a green dashed line.

Unfortunately, all efforts to co‐crystallize RosA with SAM, SAH, AF or combinations of these compounds and cofactors were unsuccessful. Therefore, we used structural bioinformatics methods to locate putative binding sites for the substrate and cofactor. A cavity analysis using the program casox 19 yielded several cavities, but only one of those was large enough to accommodate AF and SAM, and was located at the interface between the Rossmann domain and the N‐terminal domain (Fig. 3A).

**Figure 4:**
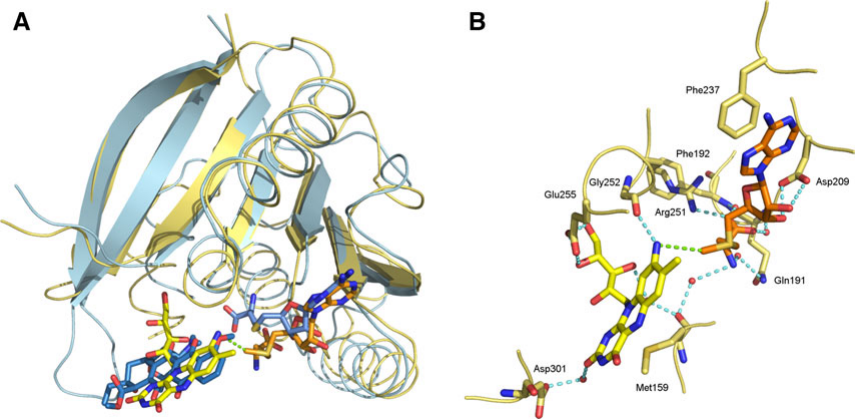
Structure of the putative active site of RosA. (A) Close‐up view of the superimposed structures of RosA (shown in yellow) and carminomycin‐4‐*O*‐methyltransferase (PDB ID 1TW2; shown in blue). Ligands of 1TW2 (*S*‐adenosylhomocysteine and 4‐methoxy‐E‐rhodomycin) are shown as blue sticks. AF and SAM are shown in their predicted binding modes as yellow and orange sticks, respectively. (B) Close‐up view of the predicted binding pocket of RosA. Potential hydrogen bonding with AF (shown in yellow) and SAM (shown in orange) is indicated by blue dashed lines. Water molecules are represented as red spheres. The distance between the reactive methyl group of SAM and the amino group of AF is indicated by a green dashed line.

In addition, the web tools prosite 20, 3dligandsite 21 and coach 22 were used to predict and identify ligand binding regions in RosA. PROSITE identified a SAM binding region (residue 235–237) in the C‐terminal Rossmann domain. This finding is in line with results obtained using 3dligandsite, coach and CaSoX. 3dligandsite identified 25 crystal structures of proteins structurally related to RosA with bound SAM or SAH, and predicted 22 residues that are putatively involved in cofactor binding to RosA. On the other hand, the program coach (http://zhanglab.ccmb.med.umich.edu/COACH/) found several structures of methyltransferases in complex with various ligands, and clustered them according to a confidence score (C‐score between 0 and 1). The best‐scored cluster predicted 13 consensus SAH/SAM binding residues with a C‐score of 0.54 and a cluster size (total number of templates in a cluster) of 103. Moreover, coach generated a structure of RosA with SAM bound in the predicted active site. All other ligand–enzyme complexes had significantly lower C‐scores. Among those structures with C‐scores between 0.5 and 0.05 were those with ligands such as *N*‐acetyl serotonin (C‐score 0.10), pisatin (C‐score 0.08) and 5‐(3,3‐dihydroxypropeny)‐3‐methoxy‐benzene‐1,2‐diol (C‐score 0.05). Flavin derivatives such as AF did not occur in these clusters identified by coach. However, a cluster with an even lower C‐score of 0.02 contained structures of methyltransferases with bound SAH and 4‐methoxy‐E‐rhodomycin. 4‐methoxy‐E‐rhodomycin resembles AF with regard to the planar ring system. A comparison of the modelled RosA–SAM complex with the structure of carminomycin 4‐*O*‐methyltransferase from *Streptomyces peucetius* (PDB ID 1TW2), a representative of the 4‐methoxy‐E‐rhodomycin cluster, showed that, despite the low sequence identity of 33%, the Rossmann domain and especially the β‐sheet core appeared to be structurally conserved. Therefore, we superimposed the β‐sheet core residues of RosA and 1TW2 (RMSD 0.805 Å; 42 of 42 aligned Cα atoms), and aligned a molecule of AF with 4‐methoxy‐E‐rhodomycin bound to 1TW2 (Fig. 4A). The RosA–AF–SAM complex thus generated was energy‐minimized and analysed in terms of the binding interactions of AF and SAM (Figs 3B and 4B).

### Determination of dissociation constants

**Figure 5:**
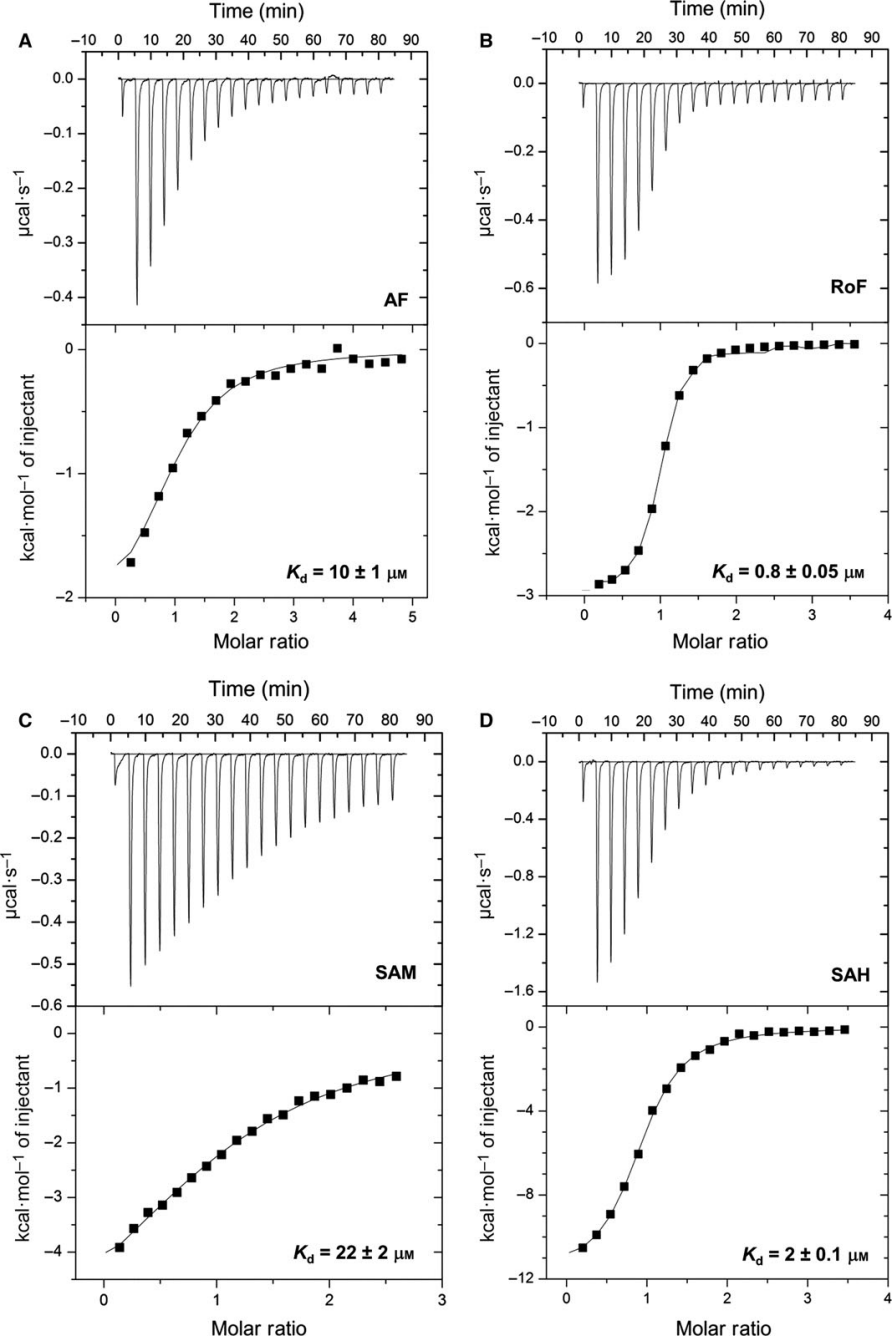
Binding of AF, RoF, SAM and SAH to RosA. Calorimetric titrations of RosA with AF (A), RoF (B), SAM (C) and SAH (D) were performed in 50 mm Tris/HCl buffer, pH 8.0, containing 100 mm NaCl at 25 °C using a VP‐ITC system (MicroCal). The dissociation constants for binding of AF, RoF, SAM and SAH to RosA were 10 ± 1, 0.8 ± 0.05, 22 ± 2 and 2 ± 0.1 μm, respectively (three independent measurements for each ligand). Data were analysed by non‐linear least‐squares fitting using origin version 7.0 (MicroCal).

Inspection of the AF and SAM binding sites in the RosA structure suggested that both substrates, AF and SAM, bind independently to their respective pockets. Thus we determined the dissociation constant for AF and SAM by isothermal titration calorimetry (ITC). As shown in Fig. 5A,C, titration with either AF or SAM produced exothermic signals that were fitted to a one‐binding‐site model, yielding dissociation constants of 10 ± 1 and 22 ± 2 μm, respectively. Surprisingly, both products, i.e. RoF and SAH, bind approximately ten times more tightly than the substrates, displaying dissociation constants of 0.8 ± 0.05 and 2 ± 0.1 μm, respectively (Fig. 5B,D).

### Ternary complex formation with substrates and products

**Figure 6:**
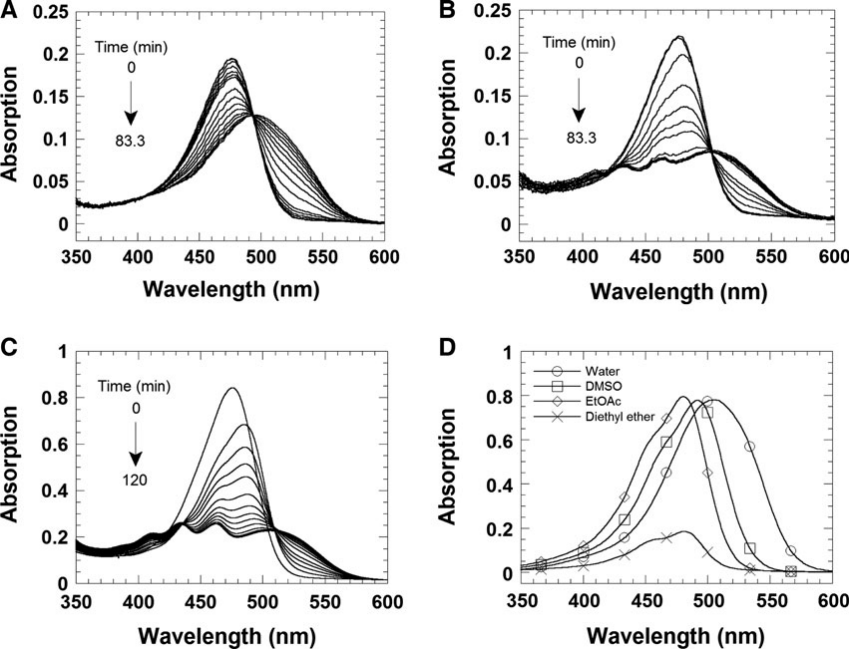
UV/Vis absorption spectra at various RosA concentrations. (A, B) Spectral changes during the reaction of 5 μm AF and 0.8 mm SAM with 5 and 25 μm RosA, respectively. The reactions were performed in 50 mm Tris/HCl buffer, pH 8.0, containing 100 mm NaCl, in a single‐mixing stopped‐flow spectrophotometer. (C) A solution of 20 μm AF and 100 μm RosA was pre‐incubated prior to addition of 2 mm SAM. All three reactions were run at 25 °C, and absorption spectra were recorded from 350 to 600 nm using a diode array. (D) Spectra for 25 μm RoF in various solvents [water, dimethylsulfoxide (DMSO) and ethyl acetate] and for 15 μm RoF in diethyl ether.

**Figure 7:**
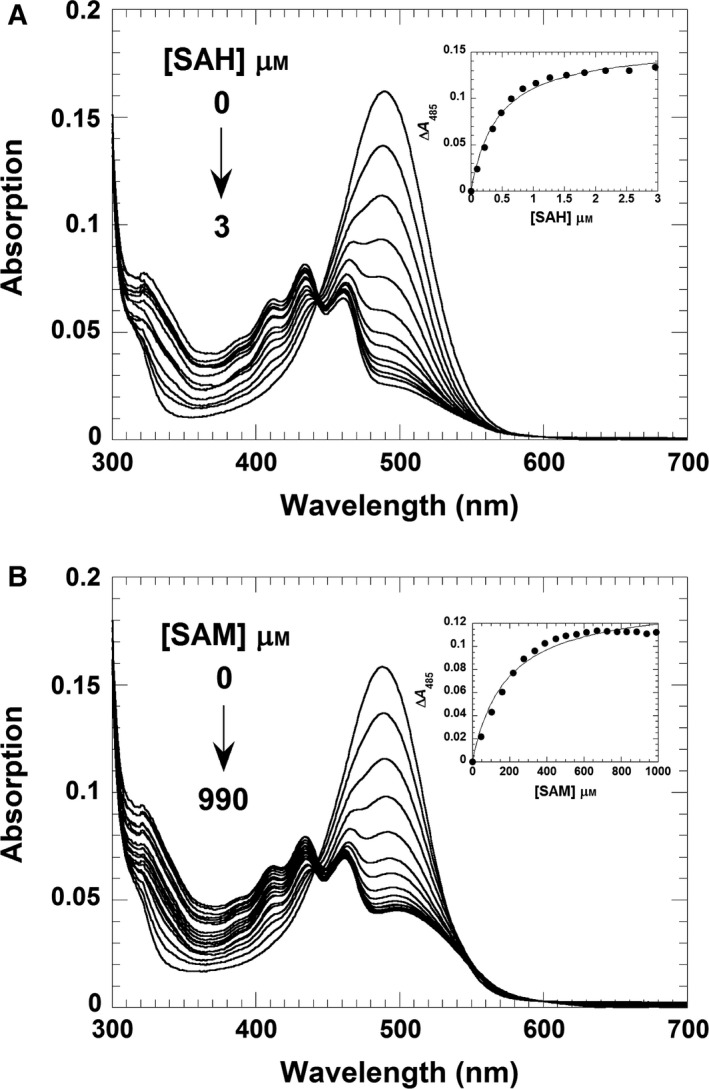
Formation of a ternary complex comprising the RosA:RoF complex with SAH or SAM. (A) A solution of RosA (25 μm) and RoF (5 μm) was titrated with SAH (0–3 μm). The absorption spectra were recorded from 300 to 700 nm using a UV/Vis spectrophotometer. The inset shows a plot of the absorption change at 485 nm as a function of SAH concentration. A hyperbolic fit to the experimental data yielded a dissociation constant of 0.4 ± 0.05 μm. (B) A solution of RosA (25 μm) and RoF (5 μm) was titrated in tandem cuvettes with SAM (0–990 μm). The inset shows a plot of the absorption change at 485 nm as a function of SAM concentration. A hyperbolic fit to the experimental data yielded a dissociation constant of 180 ± 20 μm. Both titrations were performed in 50 mm Tris/HCl buffer, pH 8.0, containing 100 mm NaCl.

The catalytic methylation of AF by RosA was investigated by spectrophotometry using a stopped‐flow device and a conventional spectrophotometer, exploiting the spectral changes that occur during methylation [λ_max_ (AF) = 478 nm; λ_max_ (RoF) = 505 nm]. At low concentrations of RosA, the final absorption spectrum features an absorption maximum at 505 nm, which is characteristic of complete formation of RoF (Fig. 6A). However, at high concentrations of RosA the maximum at 505 nm is less pronounced (hypsochromic effect), and additional maxima at a shorter wavelength are observed (Fig. 6B,C). Analysis by HPLC revealed that RoF is the main product in both cases, suggesting that the observed spectral changes are due to formation of a complex with RosA and either SAM or SAH. The observed spectral differences suggest considerable changes in the flavin binding site upon formation of a ternary complex, and the UV/Vis absorption spectra of RoF in various solvents suggest that an apolar environment induces similar spectral changes to those observed in our experiments (Fig. 6D). In addition, it is conceivable that steric restriction induced in the ternary complex prevents de‐localization of the electron lone pair into the aromatic system of the isoalloxazine ring, resulting in the observed hypso‐ and hypochromic effect. Therefore, we assume that formation of the ternary complex generates a more apolar and sterically constrained environment in the RoF binding pocket. In order to reveal the nature of the ternary complex, we also determined the affinity of RosA for SAM and SAH in the presence of RoF. As shown in Fig. 7, the presence of RoF greatly decreases the affinity for SAM, yielding a dissociation constant of 180 ± 20 μm, whereas the affinity of SAH is approximately fourfold higher (*K*_d_ = 0.4 ± 0.05 μm). Thus the ternary complex with SAH (product complex) is clearly favoured over a complex with SAM (mixed substrate/product complex).

**Figure 8:**
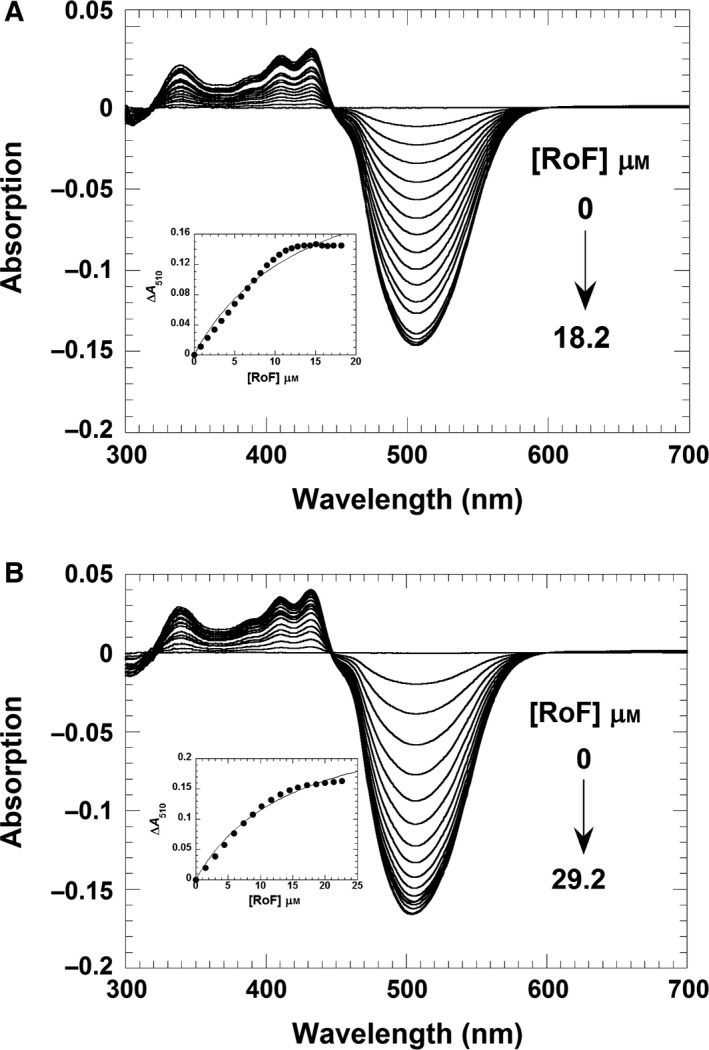
Formation of a ternary complex comprising the RosA:SAH or RosA:SAM complex with RoF. (A) A solution of RosA (25 μm) and SAH (150 μm) was titrated with RoF (0–18.2 μm). The absorption changes from the UV/Vis difference titrations were recorded from 300 to 700 nm using a UV/Vis spectrophotometer. The inset shows a plot of the absorption change at 510 nm as a function of RoF concentration. A hyperbolic fit to the experimental data yielded a dissociation constant of 14 ± 2 μm. (B) A solution of RosA (30 μm) and SAM (2 mm) was titrated in tandem cuvettes with RoF (0–29.2 μm). The inset shows a plot of the absorption change at 510 nm as a function of the RoF concentration. A hyperbolic fit to the experimental data yielded a dissociation constant of 15 ± 2 μm. Both titrations were performed in 50 mm Tris/HCl buffer, pH 8.0, containing 100 mm NaCl.

**Figure 9:**
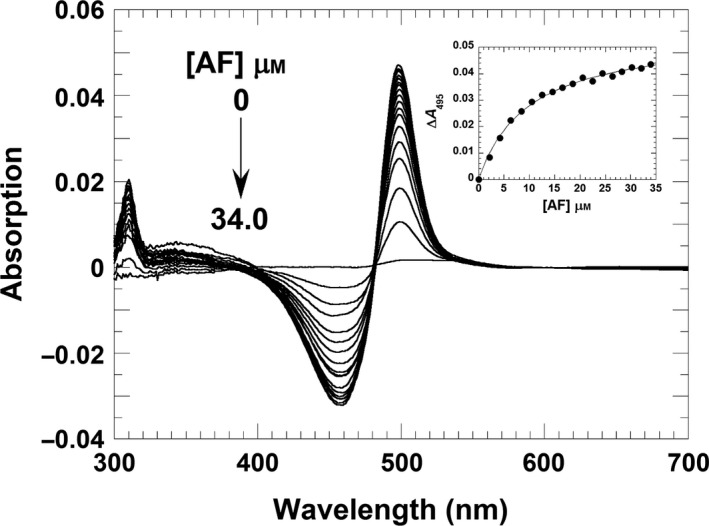
AF binds independently to RosA in the presence of SAH. A solution of RosA (25 μm) and SAH (150 μm) was titrated with AF (0–34.0 μm). The absorption spectra of the titrations were recorded from 300 to 700 nm using a UV/Vis spectrophotometer. The inset shows a plot of the absorption change at 495 nm as a function of SAH concentration. A hyperbolic fit to the experimental data yielded a dissociation constant of 10 ± 1 μm.

Next, we performed difference absorption spectroscopy to determine the dissociation constants of RoF from the binary complex of RosA:SAH and RosA:SAM, respectively, as well as the affinity of AF for the RosA:SAH complex. The former experiments, shown in Fig. 8A,B, yielded virtually identical dissociation constants of 14 ± 2 and 15 ± 2 μm, demonstrating that SAH and SAM cause similar decreases in the binding affinity of RoF by a factor of 17–18. In contrast to product binding, AF binding to RosA is not affected by SAH, as the determined dissociation constant of 10 ± 1 μm is almost identical to that observed in the absence of SAH (Fig. 9). Table 3 lists the dissociation constants obtained by ITC or spectrophotometric titrations.

**Table 3:** Dissociation constants (*K*_d_, μm) of RosA for AF, RoF, SAH and SAM, determined by ITC and UV/Vis absorption titrations. Values marked with asterisk were obtained from ITC measurements. Values marked with hash symbols (#) were obtained from UV/Vis absorption titrations

In the presence of	AF	RoF	SAM	SAH
–	10 ± 1*	0.8 ± 0.05*	22 ± 2*	2 ± 0.1*
RoF			180 ± 20^#^	0.4 ± 0.05^#^
SAM		15 ± 2^#^		
SAH	10 ± 1^#^	14 ± 2^#^		

### Mechanistic studies of the dimethylation reaction catalysed by RosA

**Scheme 2:**
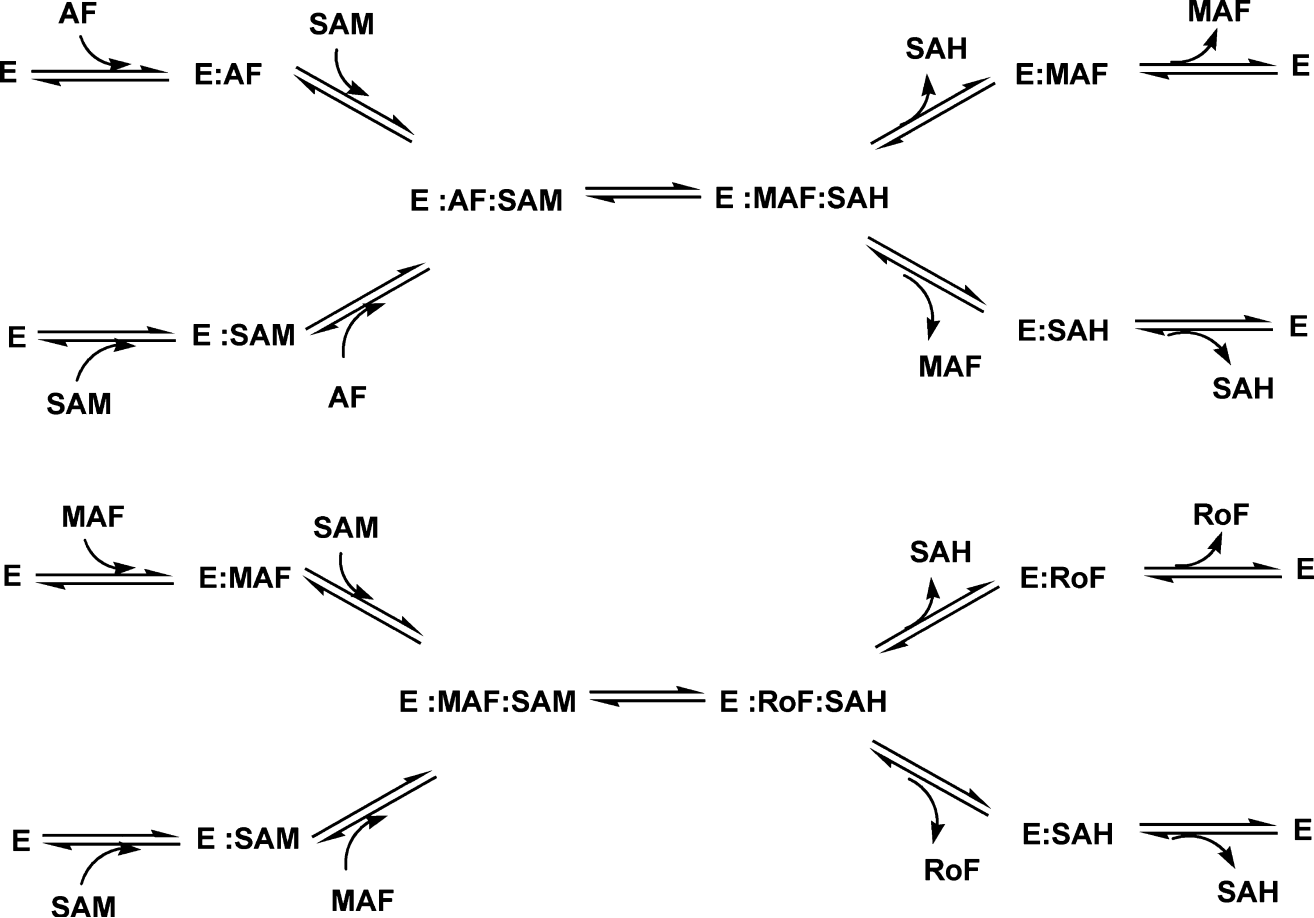
Scheme of the methylation reactions catalysed by RosA following a random‐order ‘bi‐bi’ mechanism.

**Figure 10:**
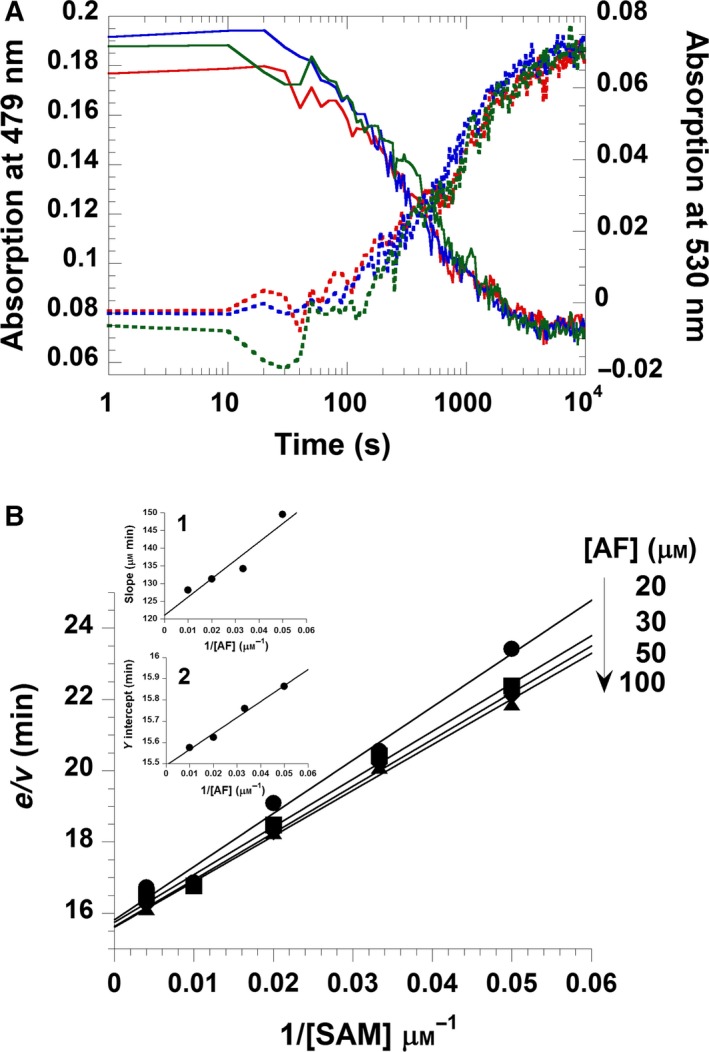
Steady‐state kinetics of RosA and substrate binding sequence of the RosA reaction. (A). The RosA reaction was performed at 25 °C in a stopped‐flow spectrophotometer using different mixing orders for the substrates AF and SAM. The traces shown represent AF consumption and RoF formation, respectively, at 479 nm (solid lines) and 530 nm (dotted lines). The red trace represents the reaction of RosA:AF (5 μm RosA and 5 μm AF) with 80 μm SAM. The blue trace represents the reaction of RosA:SAM (5 μm RosA and 80 μm SAM) with 5 μm AF. The green trace represents the reaction of RosA (5 μm) with AF (5 μm) and SAM (80 μm). All solutions were prepared in 50 mm Tris/HCl buffer, pH 8.0, containing 100 mm NaCl. (B) Double‐reciprocal plot of the two‐substrate kinetics of the RosA reaction. The enzyme catalytic assay was performed at 25 °C in a spectrophotometer, measuring the increase in RoF formation at 530 nm. The reaction contained 5 μm RosA in 50 mm Tris/HCl buffer, pH 8.0, containing 100 mm NaCl plus various concentrations of AF (20–100 μm) and SAM (20–250 μm) as indicated. Insets 1 and 2 represent secondary plots of the slope and *y*‐axis intercept obtained from the primary plot against reciprocal AF concentration, resulting in a $K_{m}^{\mathrm{AF}}$ of 4 μm, a $K_{m}^{\mathrm{SAM}}$ of 70 μm, and a *k*_cat_ of 0.06 min^−1^.

With regard to the reaction mechanism, the structure of RosA as well as the independent binding of AF and SAM suggest that the enzyme operates by a random‐order kinetic mechanism, as shown in Scheme 2. To test this hypothesis, we performed a series of pre‐steady‐state kinetic measurements in the stopped‐flow device. In these experiments, different mixing orders were employed, and the progress of the reaction was monitored at 479 and 530 nm. As shown in Fig. 10A, the rate of reaction was independent of the experimental set‐up, i.e. whether RosA was directly mixed with AF and SAM or pre‐incubated with either of the substrates. To obtain further insight into the kinetic mechanism, we performed a set of steady‐state experiments at various AF and RoF concentrations. As shown in Fig. 10B, a primary reciprocal plot of the reaction velocity against the reciprocal SAM concentration resulted in a set of converging lines in accordance with a random‐order mechanism. From a re‐plot of the primary data, i.e. slopes and *y*‐axis intercepts versus the reciprocal AF concentration (Fig. 10B, insets), the Michaelis constants of AF and SAM were deduced to be 4 and 70 μm, and the *k*_cat_ was estimated as 0.06 min^−1^ (Table 4).

**Table 4:** Kinetic parameters for the RosA reaction

Parameter	Value
$K_{m}^{\mathrm{AF}}$ (μm)	4
$K_{m}^{\mathrm{SAM}}$ (μm)	70
*k*_cat_ (min^−1^)	0.06

**Figure 11:**
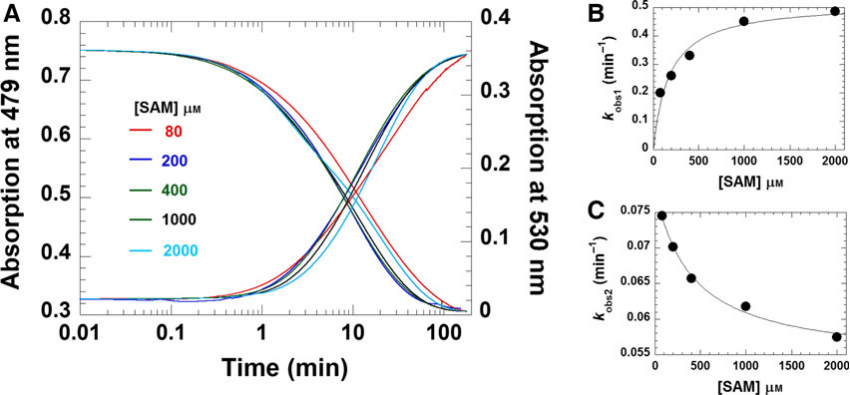
Kinetic reaction of RosA:AF (1 : 1 ratio) with SAM. (A) Reaction of RosA:AF with SAM. A solution of RosA (20 μm) and AF (20 μm) in 50 mm Tris/HCl, pH 8.0, containing 100 mm NaCl, was mixed with various concentrations of SAM (80–2000 μm), and the reaction was monitored by the absorption change at 25 °C on a spectrophotometer. All concentrations are shown as final concentrations. The absorption decrease at 479 nm (left *y*‐axis) and increase at 530 nm (right *y*‐axis) are shown. (B) *k*_obs_ values for the first phase of the reaction representing monomethylation step were plotted as a function of concentration of SAM, resulting a rate constant of 0.5 ± 0.05 min^−1^ with a *K*_d_ of 180 ± 40 μm. (C) Plot of *k*_obs_ for the second phase against the concentration of SAM. The plot was fitted by non‐linear curve fitting, consistent with a rate constant of 0.05 ± 0.002 min^−1^.

**Figure 12:**
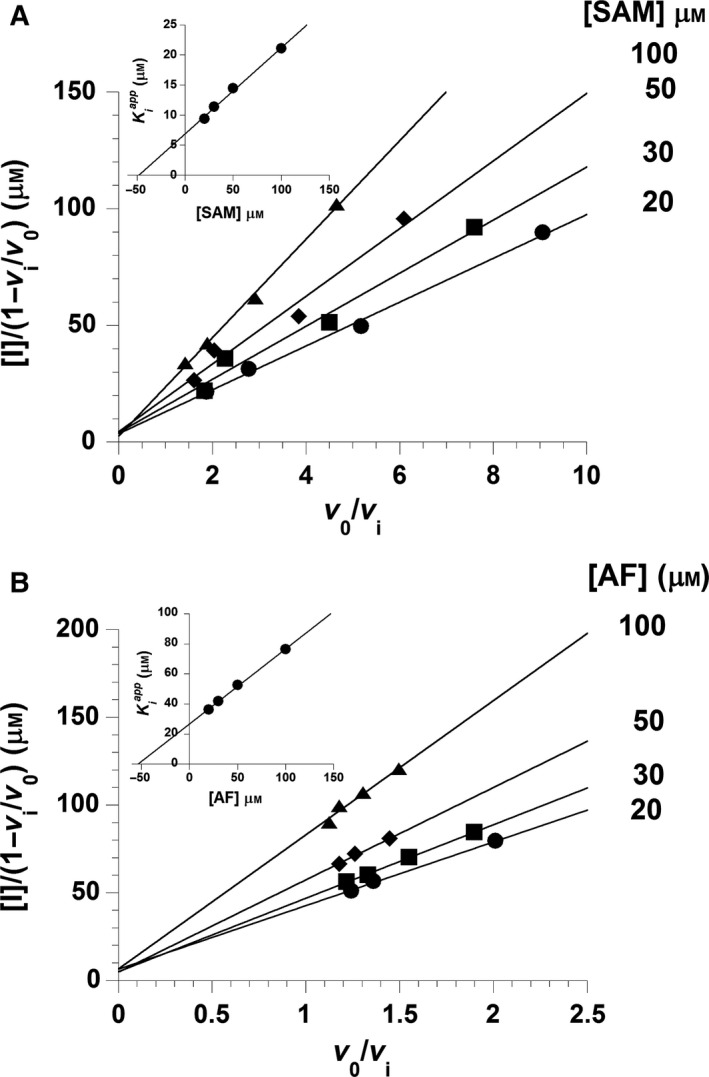
Product inhibition of the RosA reaction. (A) SAH inhibition of the RosA reaction. A Henderson plot for tight‐binding inhibition by SAH with respect to SAM was obtained by plotting [I]/(1 – *v*_i_/*v*_0_) as a function of *v*_0_/*v*_i_ at various fixed concentrations of SAM as indicated. Initial rates of RosA reactions were measured by the absorption change at 530 nm over time. The reactions contained 5 μm RosA, 200 μm AF, various fixed SAM concentrations (20–100 μm), and various concentrations of SAH (0, 10, 20, 40 and 80 μm). The inset is a secondary plot of $K_{i}^{\mathrm{app}}$, slopes obtained from the primary plot, against the concentration of SAM yielding a $K_{i}^{\mathrm{app}}$ of 7 μm. (B) RoF inhibition of the RosA reaction. A Henderson plot for tight‐binding inhibitor of product inhibition by RoF with respect to AF was obtained by plotting [I]/(1 – *v*_i_/*v*_0_) as a function of *v*_0_/*v*_i_ at various fixed concentrations of AF as indicated. Initial rates of RosA reactions were measured by the absorption change at 530 nm by time. The reactions consisted of 5 μm RosA, 250 μm SAM, various fixed AF concentrations (20–100 μm), and various concentrations of RoF (0, 10, 15, 25 and 40 μm). The inset is a secondary plot of $K_{i}^{\mathrm{app}}$, slopes obtained from the primary plot, against concentration of AF, yielding a $K_{i}^{\mathrm{ROF}}$ of 27 μm.

As generation of RoF from AF requires two consecutive methylation steps, we also wished to obtain insight into the individual methylation reactions. Towards this aim, we analysed the reaction under single‐turnover conditions at two different wavelengths that represent the consumption of the substrate (λ = 479 nm) and the generation of the product (λ = 530 nm). The reaction of RosA (20 μm) in the presence of AF (20 μm) was investigated by mixing with various concentrations of SAM (80–2000 μm). The rates of the observed spectral changes at 479 and 530 nm were biphasic, and therefore fitted to two rate equations (Fig. 11A). The first phase consisted of a decrease in absorption at 479 nm and a concurrent small increase at 530 nm (approximately 25%). This first phase was interpreted to represent monomethylation of AF to MAF, and showed a hyperbolic dependence on the SAM concentration, yielding a limiting rate of 0.5 ± 0.05 min^−1^ and an equilibrium constant of 180 ± 40 μm (Fig. 11B). The second slower phase corresponded to a decrease at 479 nm and a concomitant increase at 530 nm. This phase showed an inverse dependence on the SAM concentration approaching 0.05 ± 0.002 min^−1^ (Fig. 11C). The latter result suggested that monomethylation of AF generates an inhibitory product that prevents binding of SAM. In fact, our binding studies have revealed that SAH binds much more strongly to RosA than SAM does (Table 3). Furthermore, the presence of RoF further weakens SAM binding but the affinity for SAH is increased (Table 3). Thus we reasoned that SAH, which is produced in the first monomethylation reaction, acts as a competitive inhibitor of SAM. To test this hypothesis, we determined the rate of the methylation reaction as a function of SAM and SAH concentrations. The initial velocities were determined as a function of SAM concentration at various fixed concentrations of SAH to obtain information on the type of inhibition. However, this plot did not allow us to discern the inhibition type, i.e. competitive, non‐competitive or uncompetitive. As it was shown that SAH binds more tightly to RosA than SAM does, we used the linearized Henderson equation for data analysis 23. The resulting Henderson plot, as shown in Fig. 12A, yielded a set of lines that converge on the *y* axis. A re‐plot of the slopes, as shown in the inset to Fig. 12A, allows determination of the inhibition constant of SAH as 7 μm. This value is in the same range as the dissociation constant of SAH in the presence of RoF, suggesting that MAF and RoF have similar effects on SAH binding (Table 3). Thus we conclude that the observed rate decline of the second kinetic phase is due to the competitive effect of SAH, with the limiting value corresponding to *k*_off_ of SAH.

In a similar set of experiments, it was shown that RoF is a competitive inhibitor of AF, exhibiting a *K*_i_ value of 27 μm (Fig. 12B), which represents the dissociation constant of RoF in the presence of SAM (compare Tables 3 and 5).

**Table 5:** Product inhibition pattern and dissociation constants (*K*_i_) of the RosA–product inhibitor complex. Product inhibition was fitted to the tight‐binding inhibitor model

Product	Type of inhibition	*K*_i_ (μm)
RoF	Competitive (AF)	27
SAH	Competitive (SAM)	7

## Discussion

Structure analysis revealed that RosA adopts a topology that is characteristic of SAM‐dependent methyltransferases. Although attempts to obtain structural information for complexes with AF, SAM and SAH were unsuccessful, further inspection of the active site and the AF and SAM binding pockets provided useful hints for mechanistic considerations that were tested by binding studies and steady‐state as well as pre‐steady‐state kinetic experiments. The final biosynthesis of RoF from AF by RosA is unusual in the sense that the enzyme performs two consecutive methylations in the same active site, and hence SAH must dissociate to make room for another SAM while the monomethylated intermediate, MAF, may remain bound to the flavin‐binding pocket. This complexity of the overall conversion of AF to RoF was analysed by binding studies as well as kinetic experiments to evaluate the interaction of substrates and products in the RosA‐catalysed reaction. Much to our surprise, our binding studies revealed that both products, RoF and SAH, bind approximately ten times more tightly to RosA than AF and SAM do (Table 3). Interestingly, the affinity of RoF decreased approximately 20‐fold in the presence of SAM and SAH, whereas the affinities of SAM and SAH respond differently to the presence of RoF, i.e. the dissociation constant for SAM increased approximately eightfold and that for SAH decreased fourfold. These relative binding affinities clearly suggest that the ternary complex observed in spectrophotometric experiments (Fig. 6) consisted of RoF and SAH bound to RosA. The fact that the tightest binary complex formed is that between RosA and RoF also suggested a potential mechanism to control the concentration of free RoF in *S. davawensis*. It may be assumed that the concentrations of both SAM and SAH are too low to form a ternary complex with RosA:RoF, and thus RoF inhibits binding of AF and further generation of the product. This scenario is also supported by the competitive inhibition of the RosA‐catalysed methylation of AF by RoF (Fig. 12). The resistance of *S. davawensis* to RoF appears to be related to the ability of the *ribB* FMN riboswitch to discriminate between FMN and roseoflavin‐5’‐phosphate 7. However, it is not clear yet whether other additional mechanisms contribute to resistance in *S. davawensis*. Therefore, it is plausible that the tight binding of RoF to RosA constitutes an additional mechanism to confer resistance towards RoF. Although it is still unknown how RoF is eventually secreted, it is conceivable that RosA also serves as a chaperone to guide RoF to the secretion machinery that is in charge of its translocation from the cell into the environment.

**Scheme 3:**
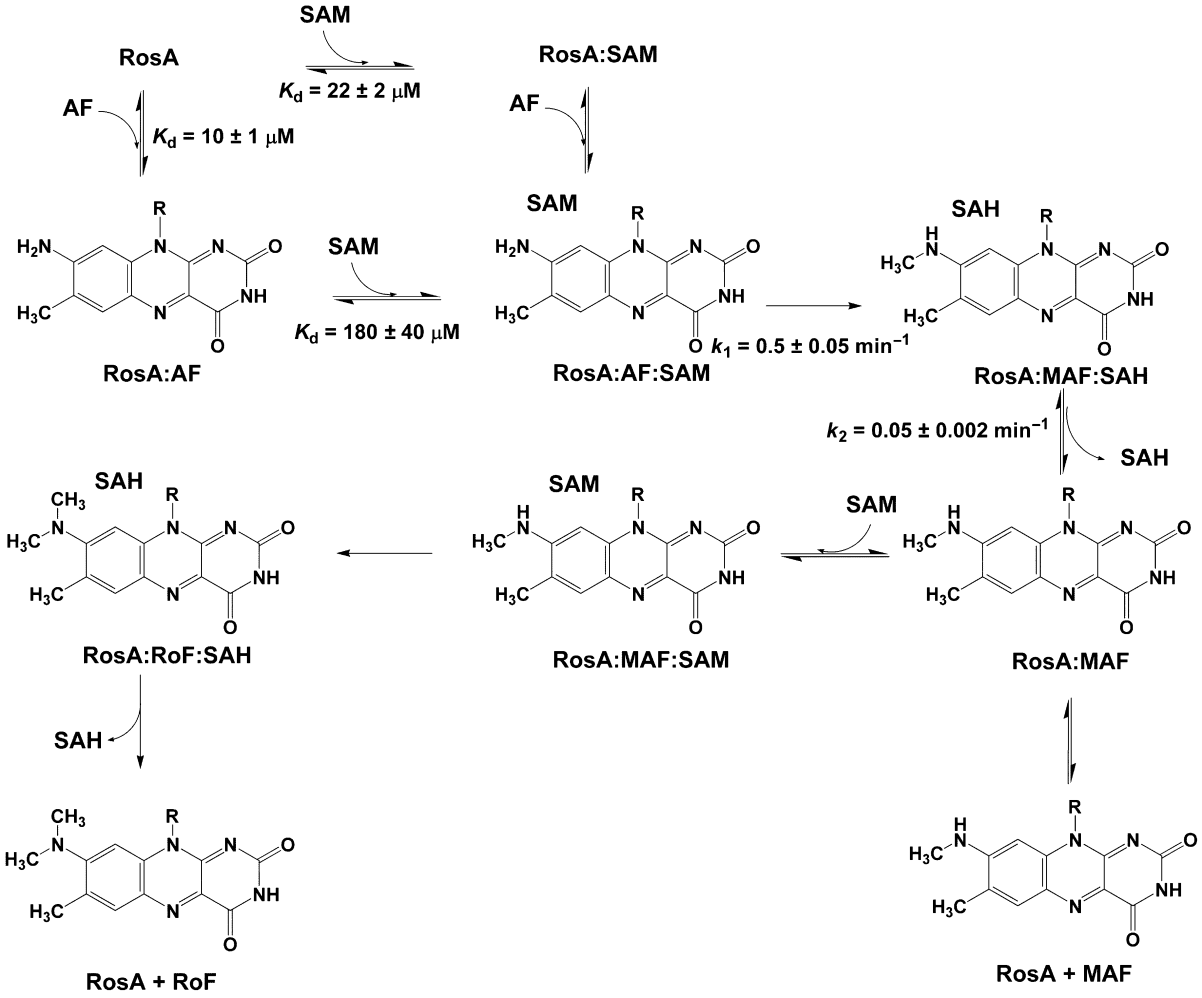
Suggested reaction scheme for the reactions catalysed by RosA based on our binding studies and kinetic measurements.

The X‐ray crystallographic structure of RosA suggested that both substrates, AF and SAM, bind independently to their respective pockets. This feature was confirmed by kinetic experiments that demonstrated a random‐order binding mechanism for the first methylation reaction yielding MAF and SAH (Fig. 10 and Scheme 3). The rate of monomethylation of AF to MAF showed a hyperbolic dependence on the SAM concentration, yielding a limiting rate of 0.5 ± 0.05 min^−1^ (Fig. 11B). Surprisingly, the subsequent reaction phase was characterized by an inverse dependence on the SAM concentration, approaching a limiting rate of 0.05 ± 0.002 min^−1^ (Fig. 11C). As noted above, SAH binds tightly to RosA, and its affinity is enhanced in the presence of RoF (Table 3). It is likely that the binding affinity of SAH in the presence of MAF is in the same range, i.e. between 2 ± 0.1 and 0.4 ± 0.05 μm, and thus the second methylation reaction depends on the rate of dissociation of SAH (*k*_off_). This *k*_off_ appears to be the limiting step for conversion of MAF to RoF, and therefore the intrinsic rate for this reaction step is not accessible in our kinetic experiments. A summary of the reaction steps characterized in this study is shown in Scheme 3.

## Experimental procedures

### Reagents

All chemicals and reagents were of the highest purity commercially available from Sigma‐Aldrich (St. Louis, MO, USA) and Merck (Darmstadt, Germany). AF was a generous gift from Sandro Ghisla (Universität Konstanz, Germany). Ni‐NTA‐agarose was obtained from GE Healthcare (Little Chalfont, UK). RosA was over‐produced in *Escherichia coli*, and purified as previously described 11. The concentrations of the following compounds were determined spectrophotometrically using these extinction coefficients: AF, ε_490_ = 42.0 mm ^−1^·cm^−1^; RoF, ε_505_ = 31.1 mm ^−1^·cm^−1^ 24; SAM and SAH, ε_260_ = 15.4 mm ^−1·^cm^−1^ 25; RosA (based on amino acid sequence) = 27.1 mm ^−1·^cm^−1^.

### Expression and purification

Heterologous production and purification of recombinant hexahistidine‐tagged RosA from *S. davawensis* was performed as described previously 11. *E. coli* Rosetta 2 (DE3) was transformed using pET24a(+)*rosA‐His*_*6*_ (pFJ02) 11. One of the transformant strains was used to inoculate a pre‐culture that was aerobically incubated at 37 °C for 16 h (200 rpm) in lysogeny broth containing 50 μg·mL^−1^ kanamycin. The cells were harvested by centrifugation (5000 ***g*** at 4 °C for 30 min), and washed three times using PBS (10 min per wash at 4 °C). The cells were then suspended in 30 mL PBS and used as an inoculum for a 15‐L bioreactor (Bioengineering AG, Wald, Switzerland). Cell growth was allowed to proceed for 7 h at 750 rpm, 37 °C, with an aeration of 1 L of oxygen per L of culture per minute in a total volume of 10 L until the cells reached an absorption at 600 nm of 0.8. Synthesis of the recombinant protein was induced by addition of 0.5 mm isopropyl‐β‐d‐thiogalactopyranoside. The culture was further incubated for 16 h at 30 °C until an attenuance at 600 nm of 1.2 was reached. Cells were harvested by centrifugation at 5000 ***g*** (4 °C).

To produce selenomethionine‐containing hexahistidine‐tagged RosA, the expression protocol for native RosA was slightly altered. *E. coli* BL834 (DE3) was transformed with pFJ02, and incubated for 7 h at 37 °C and 200 rpm in selenomethionine minimal medium 26 supplemented with 50 μg·mL^−1^ kanamycin. The pre‐culture was harvested by centrifugation at 5000 ***g*** (4 °C) and used as an inoculum for a 15‐L bioreactor (Bioengineering AG). Synthesis of the recombinant protein was induced at when the cells reached an attenuance at 600 nm of 0.8 by adding 0.5 mm isopropyl‐β‐d‐thiogalactopyranoside. Cells were harvested when they reached an attenuance at 600 nm of 1.2, by centrifugation at 5000 ***g*** (4 °C). All cell pellets were frozen immediately after harvesting and stored at −80 °C.

The enzyme was purified using an ÄKTApurifier^™^ system (GE Healthcare). Frozen cell pellets of *E. coli* Rosetta 2 (DE3) and BL834 (DE3) over‐producing hexahistidine‐tagged RosA and SeMet‐RosA, respectively, was resuspended in 50 mL HisTrap binding buffer. This buffer contained 20 mm Na_2_HPO_4_, pH 7.4, 500 mm NaCl and 20 mm imidazole, and was supplemented with protease inhibitors (Roche cOmplete^™^ EDTA‐free protease inhibitor cocktail, Roche, Basel, Switzerland). For purification of SeMet‐RosA, the HisTrap buffer also contained 2 mm β‐mercaptoethanol. The cells were disrupted using a French press (three cycles, 2 × 10^8^ Pa, 10 °C). The cell‐free extract was cleared by centrifugation at 10 000 ***g*** for 30 min at 4 °C. The supernatant was again centrifuged at 100 000 ***g*** for 30 min at 4 °C to remove cell debris and unbroken cells. The cleared lysate was filtered (0.45 μm), equilibrated with loading buffer (20 mm Na_2_HPO_4_, pH 7.4, 500 mm NaCl, 20 mm imidazole, plus 2 mm β‐mercaptoethanol for SeMET‐RosA) and applied to a 5 mL HisTrap column (HisTrap^™^ HP; GE Healthcare). Gradient elution of the hexahistidine‐tagged protein was performed using 0 to 50% elution buffer (20 mm Na_2_HPO_4_, pH 7.4, 500 mm NaCl, 500 mm imidazole, plus 2 mm β‐mercaptoethanol for SeMET‐RosA). Eluted enzyme fractions were pooled and desalted using a HiTrap^™^ desalting column (GE Healthcare), and equilibrated in a buffer containing 20 mm Tris/HCl, pH 8.0, with 2 mm β‐mercaptoethanol for SeMET‐RosA only. To achieve enzyme purity appropriate for crystallization experiments, an additional ion exchange chromatography step was performed. The enzyme fraction was applied to a MonoQ 5/50 GL column (GE Healthcare) and eluted using a linear gradient from 0 to 100% elution buffer comprising 20 mm Tris/HCl, pH 8.0, 500 mm NaCl (plus 2 mm β‐mercaptoethanol for SeMET‐RosA only). Aliquots of the fractions were analysed by SDS/PAGE with staining using Coomassie Brilliant Blue R‐250. The homogeneous fractions were tested directly for RosA activity (see below).

### Determination of native and subunit molecular masses of RosA

To determine the subunit molecular mass of RosA purified protein was loaded onto SDS/PAGE (12.5%). Protein molecular mass markers used were Low Molecular Weight protein markers (GE Healthcare, Little Chalfont, UK) containing phosphorylase b (97 kDa), albumin (66 kDa), ovalbumin (45 kDa), carbonic anhydrase (30 kDa), trypsin inhibitor (20 kDa), and α‐lactalbumin (14.4 kDa). The gel was stained with Coomassie Brilliant Blue R‐250 prior to destaining (10% ethanol, 10% glacial acetic acid in water).

To determine the native molecular mass of RosA gel filtration chromatography at 25 °C was performed. The protein solution was loaded onto a Superdex 200 10/300 GL column attached to a AKTApurifier^™^ system (GE Healthcare) and then eluted with elution buffer (50 mm Tris‐HCl, pH 8.0 containing 100 mm NaCl) at a flow rate of 0.5 mL·min^−1^. Protein standard markers used in this experiment were ferritin (440 kDa), aldolase (158 kDa), conalbumin (75 kDa), ovalbumin (43 kDa), and ribonuclease a (13.7 kDa).

### Isothermal titration calorimetry (ITC)

All of the experiments to determine dissociation constants (*K*_d_) for AF, RoF, SAM and SAH to RosA were performed at 25 °C in 50 mm Tris/HCl buffer, pH 8.0, containing 100 mm NaCl using a VP‐ITC system (MicroCal, Northampton, MA, USA). All solutions were degassed before measurements. Titration experiments for AF and RoF binding to RosA (30 μm, 1.42 mL) consisted of 20 injections (15 μL; duration, 30 s; spacing time, 250 s) of a solution containing 0.6 mm AF and 0.48 mm RoF. Determination of the *K*_d_ for SAM and SAH was performed by titration (20 injections) of 0.35 mm SAM (15 μL; duration, 30 s; spacing time, 250 s) and 0.48 mm SAH (15 μL; duration, 30 s; spacing time, 250 s) to a RosA solution (30 μm, 1.42 mL). The ITC raw data were analysed using origin version 7.0 (MicroCal).

### Spectrophotometric titration of RosA with AF, RoF, SAM and SAH

UV/Vis absorption spectra were recorded using a Specord 200 Plus spectrophotometer (Analytik Jena, Jena, Germany) at 25 °C. To determine the dissociation constants for binding of RoF and AF to the RosA:SAH complex by means of difference spectrophotometry, titrations were performed at 25 °C in tandem cuvettes. All solutions were prepared in 50 mm Tris/HCl buffer, pH 8.0, containing 100 mm NaCl. A solution (0.8 mL) containing RosA (25 μm) and SAH (150 μm) was placed in one of the two chambers of the sample and reference cell. The other chamber of each tandem cuvette was filled with the same volume of buffer only. After recording a baseline, RoF (0–18.2 μm) or AF (0–34.0 μm) were added to the solution containing RosA and SAH in the sample cell and to the buffer in the reference cell at 2 min intervals. Absorption spectra were recorded from 300 to 700 nm after each titration step. Similar titrations of the RosA:SAM complex (30 μm RosA and 2 mm SAM) with RoF were performed by addition of RoF (0–29.2 μm) to the complex in the sample cell and to the buffer in the reference cell. For determination of the dissociation constants for SAH and SAM from the binary RosA:RoF complex (25 μm RosA and 5 μm RoF), SAH and SAM were titrated to final concentrations of 3 and 990 μm, respectively. Absorption changes were recorded from 300 to 700 nm, and were plotted against the concentration of ligands. The dissociation constants were obtained by fitting the data using Levenberg–Marquardt algorithm (Eqn (1)) implemented in the kaleidagraph software (Synergy Software, Reading, PA, USA):

(1) $$\Delta A=\frac{\Delta A_{\max}\left[L\right]}{K_{d}+\left[L\right]}$$

### Steady‐state kinetics of RosA

RosA catalyses the *N*,*N*‐dimethylation of AF to yield RoF as the final product. Steady‐state kinetic measurements were performed at 25 °C using a spectrophotometer. The assay reaction contained 5 μm RosA, various concentrations of AF (20–100 μm), and various concentrations of SAM (20–250 μm) in 50 mm Tris/HCl buffer, pH 8.0, containing 100 mm NaCl. Progress of the RosA reactions was monitored at 530 nm (ε_530_ = 25.2 mm ^−1^·cm^−1^), at which wavelength RoF formation may be monitored without interference from other chromophores. Initial rates were obtained using kinetic studio software (TgK Scientific Ltd, Bradford‐on‐Avon, UK). A double‐reciprocal plot of the initial rate and the substrate concentration provides the steady‐state kinetic parameters, and also provides information on the mechanism of a two‐substrate enzyme reaction according to Dalziel's equation (Eqn (2)), in which *e*/*v* is the ratio between the enzyme concentration and the initial velocity, [A] and [B] are the concentrations of substrates A and B, respectively, and Φ values are Dalziel's coefficients 27:

(2) $$\frac{e}{v}=\Phi_{0}+\frac{\Phi_{A}}{\left[A\right]}+\frac{\Phi_{B}}{\left[B\right]}+\frac{\Phi_{\mathrm{AB}}}{\left[A][B\right]}$$

### Product inhibition of RosA

Enzymatic activity of RosA in the presence of the reaction products RoF and SAH was studied by measuring the initial rate of the reaction at 25 °C using a spectrophotometer. The assay reaction contained 5 μm RosA, various fixed concentrations of AF (20–100 μm) and various fixed concentrations of SAM (20–100 μm) in 50 mm Tris/HCl buffer, pH 8.0, containing 100 mm NaCl. Concentrations of RoF in the range of 0–40 μm were used with various concentrations of AF and a fixed concentration of SAM (250 μm). The inhibition by SAH was analysed at various concentrations of SAH and SAM using a fixed concentration of AF (200 μm). Initial rates were obtained from linear data fitting using kinetic studio software (TgK Scientific Ltd). A Henderson plot of [I]/(1 – *v*_i_/*v*_0_) and the reciprocal of the fractional initial velocity (*v*_0_/*v*_i_) (Eqns (3) and (4)) was used to obtain inhibition constants and information on the inhibition mechanism 23:

(3) $$\frac{\left[I\right]}{1-\frac{v_{i}}{v_{0}}}=K_{i}^{\mathrm{app}}(\frac{v_{0}}{v_{i}})+\left[E\right]$$

and for a competitive inhibitor:

(4) $$K_{i}^{\mathrm{app}}=K_{i}(1+\frac{\left[S\right]}{K_{m}})$$

In Eqns (3) and (4), [I] is the concentration of inhibitor (i.e. RoF and SAH), *v*_0_ and *v*_i_ are the initial velocity of a reaction in the absence and presence of a inhibitor, respectively, [E] is the total enzyme concentration, [S] is the variable substrate concentration, *K*_m_ is the Michaelis constant, *K*_i_ represents the inhibition constant of a tight binding inhibitor and $K_{i}^{\mathrm{app}}$ represents the apparent inhibition constant.

### Pre‐steady‐state reaction studies

The methylation of AF bound to RosA was investigated at various concentrations of SAM at 25 °C using a spectrophotometer. Briefly, a solution of RosA (20 μm) and AF (20 μm) in 50 mm Tris/HCl buffer, pH 8.0, containing 100 mm NaCl was pre‐incubated at 25 °C for 3 min, and then the reaction was initiated by adding SAM (final concentrations of 80, 200, 400, 1000 and 2000 μm). Progress of the reaction due to consumption of AF and formation of the final product (RoF) was monitored at 479 and 530 nm, respectively. Data analysis of the kinetic traces was performed using the exponential equations from kinetic studio software (TgK Scientific Ltd) to obtain the observed rates (*k*_obs_). Rate constants were determined from plots of *k*_obs_ as a function of the SAM concentration using the Levenberg–Marquardt algorithm implemented in the kaleidagraph software (Synergy Software).

### Stopped‐flow spectrophotometric studies

All experiments were performed at 25 °C using an SF‐61SX2 stopped‐flow spectrophotometer (TgK Scientific Ltd, Bradford‐on‐Avon, UK) with a 1 cm path length. Prior to all experiments, the stopped‐flow instrument was flushed with 50 mm Tris/HCl buffer, pH 8, containing 100 mm NaCl several times. To analyse the effect of substrate binding order on the reaction kinetics, experiments with different mixing orders of AF and SAM were performed. Various pre‐mixed solutions of RosA, RosA:AF or RosA:SAM complexes were prepared, and then mixed with solutions containing AF and SAM, SAM or AF, respectively. The progress of the reactions was monitored at 479 and 530 nm using a KinetaScan diode array detector (TgK Scientific Ltd).

### Product analysis by HPLC

HPLC analysis of AF, MAF and RoF was performed using an Atlantis^®^ dC18 reversed‐phase column (5 μm, 4.6 × 250 mm; Waters, Milford, MA, USA) on an Ultamate 3000 HPLC instrument (Dionex, Sunnyvale, CA, USA) at 25 °C. All samples prepared from RosA reactions were loaded onto the column and eluted using a multi‐step gradient of 100 mm formic acid and 100 mm ammonium formate (pH 3.7)/methanol (0–3 min, 30% methanol; 3–20 min, 30–75% methanol; 20–22 min, 75% methanol) at a flow rate of 0.8 mL·min^−1^. Detection of AF, MAF and RoF was performed by measuring the absorption at 479, 490 and 509 nm, respectively.

### Crystallization

Crystallization drops containing purified native RosA at a concentration of 20 mg·mL^−1^ in 20 mm Tris/HCl, pH 8.0, 500 mm NaCl, were set up with commercially available screening solutions using the microbatch method on an Oryx‐7 crystallization robot (Douglas Instruments Ltd, Hungerford, UK) at 293 K. Drops were prepared by mixing equal amounts (1.0 μL) of protein and precipitant. Crystals of native RosA grew within 5 days after mixing the protein solution (in a 1 : 1 ratio) with a solution containing 200 mm sodium chloride, 100 mm Tris/HCl, pH 5.5, and 25% w/v PEG‐3350.

The same approach was used to grow crystals of the selenomethionine‐labelled derivative. Equal amounts (0.5 μL) of the variant at a concentration of 20 mg·mL^−1^ in 20 mm Tris/HCl, pH 8.0, 500 mm NaCl and 2 mm β‐mercaptoethanol were mixed with precipitant containing 100 mm MES/imidazole, pH 6.5, 20 mm d‐glucose, d‐mannose, d‐galactose, l‐fructose, d‐xylose and *N*‐acetyl‐d‐glucosamine, 10% w/v PEG‐20000 and 20% v/v PEG‐MME 550 28. SeMet‐RosA crystals grew within 7 days after crystallization set‐up. For diffraction data collection, crystals were harvested from their mother liquor using CryoLoops^™^ (Hampton Research, Aliso Viejo, CA, USA), and flash‐cooled in liquid nitrogen without any additional cryoprotection.

### Structure determination and refinement

Diffraction data to a resolution of 2.2 Å were collected for native RosA from a single triclinic crystal (space group *P*1) at beamline ID23‐1 of the European Synchrotron Radiation Facility (Grenoble, France). MAD data to a resolution of approximately 3.5 Å were collected at beamline X06DA‐PXIII of the Swiss Light Source at the Paul Scherrer Institute (Villigen, Switzerland). Three MAD datasets were collected from a single tetragonal SeMet‐RosA crystal (space group *I*4_1_22) at various wavelengths (peak, inflection, remote) that were determined from an X‐ray fluorescence scan recorded around the selenium absorption edge. Data processing and scaling for the native and SeMet MAD datasets were performed using xds 29, scala and aimless 30.

The calculated Matthews coefficient 30, 31 of the MAD datasets indicated a 99.9% probability for the presence of one RosA molecule per asymmetric unit of the crystal. To identify selenium sites, the two MAD pipelines auto‐rickshaw 32, 33 and phenix AutoSol 34, 35 were used. The combination of these programs yielded first phases and a partial structure. These phases were further used as input phases for the automated chain‐tracing/building programs phenix autobuild 36 and buccaneer 37 to extend the model and to improve its quality. The combined model of both rebuilding programs produced interpretable electron densities, with preliminary values of *R* = 37% and *R*_free_ = 49%. *R*_free_ values were computed from a set of randomly chosen reflections (5%), which were not used during refinement 38. Structure refinement was performed using the refinement programs phenix refine 34 and refmac 39. Model rebuilding was performed using buccaneer 37 and coot 40 by alternate automated rebuilding and real‐space fitting against σ_A_‐weighted 2 *F*_o_ *– F*_c_ and *F*_o_ *– F*_c_ electron density maps together with least‐squares optimization.

The improved MAD model was used as an initial molecular replacement template for phaser 41 to determine the structure of native RosA in the triclinic crystal form. Molecular replacement resulted in six molecules in the asymmetric unit. Structure refinement and model building were performed using phenix refine 34 and coot 40 by real‐space fitting against σ_A_‐weighted 2* F*_o_ *– F*_c_ and *F*_o_ *– F*_c_ electron density maps and least‐squares optimizations. Water molecules were placed into the difference electron density map, and accepted or rejected on the basis of geometry criteria as well as refined B‐factors. In later stages of the refinement, four TLS groups per protomer were defined based on an analysis using the TLSMD web server 42. The final model was refined to *R* = 20% and *R*_free_ = 24%. Validation of the structure was performed using molprobity 43, yielding a Ramachandran plot with 97.20% of the residues in favoured regions, 2.65% in allowed regions and 0.15% in disallowed regions. Data statistics and details of structure refinement are given in Table 1 (MAD data) and Table 2 (native data).

### Prediction of the SAM/SAH binding site

In order to locate putative binding sites for the substrate AF and the cofactor SAM, the program casox 19 together with the web tools prosite (sequence‐based prediction) 20, 3dligandsite (sequence‐ and structure‐based prediction) 21 and coach (sequence‐ and structure‐based prediction) 22 were used. The results of the cavity analysis using casox were compared with consensus ligand binding residues predicted by PROSITE, 3dligandsite and coach. coach additionally generated putative enzyme–ligand model complexes and ranked them according to an intrinsic C‐score. This C‐score represents a confidence score for the prediction. C‐scores range between 0 and 1, with a higher score indicating a more reliable prediction. The highest‐rated complex, together with other related methyltransferase structures, were superimposed with RosA to identify ligand binding modes. The modelled RosA–ligand complex was further energy‐minimized using the program yasara with the AMBER03 force field, employing the standard optimization protocol 44. Structure alignments were performed using pymol (https://www.pymol.org/).

## Author contributions

M.M. and P.M. initiated the project; C.T., M.K.U., M.M., K.G. and P.M. designed the experiments and analysed the data; C.T. and F.J. expressed and purified RosA; M.K.U. and K.G. crystallized RosA and determined the crystal structure; C.T. performed analytical and biochemical experiments, and determined dissociation constants as well as kinetic parameters; C.T., M.K.U., M.M., K.G. and P.M. wrote the manuscript.
